# [^99m^Tc]Tc-sestamibi SPECT/CT for the diagnosis of kidney tumours: a multi-centre feasibility study (MULTI-MIBI Study)

**DOI:** 10.1007/s00259-025-07525-3

**Published:** 2025-09-23

**Authors:** Hannah Warren, Thomas Wagner, Soha El-Sheikh, Nick Campain, Tze M. Wah, Tim S. O’Brien, Iosif A. Mendichovszky, Sabina Dizdarevic, Charlie Stewart, Helen Ng, James Blackmur, Patrick Rogers, Andrew Scarsbrook, Dhruba Dasgupta, Fahim Ul-Hassan, Nitasha Singh, Ammar Alanbuki, Maryam Jessop, Linda Park, Kelly Leonard, Alex Wood, Ben Challacombe, Grant D. Stewart, Ravi Barod, Prasad Patki, Faiz Mumtaz, Axel Bex, Veeru Kasivisvanathan, William Wildgoose, Sigrun Clark, Cecilia Vindrola-Padros, Elena Pizzo, Hakim-Moulay Dehbi, Mark Emberton, Maxine GB Tran

**Affiliations:** 1 Division of Surgery and Interventional Science, University College London, London, UK; 2 Specialist Centre for Kidney Cancer, Urology Department, Royal Free Hospital, London, UK; 3 Department of Nuclear Medicine, Royal Free Hospital, London, UK; 4 Specialist Centre for Kidney Cancer, Department of Histopathology, Royal Free Hospital, London, UK; 5 Department of Urology, Royal Devon University Hospitals NHS Foundation Trust, Exeter, UK; 6 Department of Radiology, Leeds Teaching Hospitals NHS Trust, Leeds, UK; 7 Department of Urology, Guy’s & St Thomas’ NHS Foundation Trust, London, UK; 8 Department of Nuclear Medicine, Cambridge University Hospitals NHS Foundation Trust, Cambridge, UK; 9 Department of Radiology, University of Cambridge, Cambridge, UK; 10 Department of Nuclear Medicine, University Hospitals Sussex NHS Foundation Trust, Brighton, UK; 11 Brighton and Sussex Medical School, Brighton, UK; 12 Department of Urology, Cambridge University Hospitals NHS Foundation Trust, Cambridge, UK; 13 Department of Radiology, Royal Devon University Healthcare NHS Foundation Trust, Exeter, UK; 14 Department of Nuclear Medicine, Guy’s & St Thomas’ NHS Foundation Trust, London, UK; 15 Department of Urology, University Hospitals Sussex NHS Foundation Trust, Brighton, UK; 16 Department of Surgery, University of Cambridge, Cambridge Biomedical Campus, Cambridge, UK; 17 Patient and Public representative, University College London, London, UK; 18 Rapid Research, Evaluation and Appraisal Lab (RREAL), Department of Targeted Intervention, University College London, London, UK; 19 Department of Applied Health Research, University College London, London, UK; 20 Comprehensive Clinical Trials Unit, Institute of Clinical Trials and Methodology, UCL, London, UK

**Keywords:** Renal cancer, Oncocytoma, Diagnostic accuracy, SPECT/CT, Sestamibi

## Abstract

**Purpose:**

[^99m^Tc]Tc-sestamibi SPECT/CT (MIBI SPECT/CT) is a promising tool to differentiate benign and malignant renal tumours. We tested feasibility of recruitment to a prospective, multi-centre diagnostic test evaluation study of MIBI SPECT/CT for T1 renal tumours.

**Methods:**

Consecutive adult patients with a newly-diagnosed clinical T1 (cT1) renal mass (2–7 cm) presenting to participating sites December 2022 - February 2024 were recruited and underwent MIBI SPECT/CT prior to histopathological diagnosis. Patients who accepted and declined participation and clinicians involved in study activities were invited to a semi-structured interview. The primary endpoint was feasibility of multi-centre recruitment. Secondary endpoints included qualitative assessment of barriers and facilitators to participation, estimates of MIBI SPECT/CT accuracy to detect cancer in order to power a definitive study, inter-rater agreement and identifying training needs for scan acquisition and interpretation.

**Results:**

Of 109 approached patients, 50 enrolled and underwent the study scan (45.8%, 95% CI 36.2–55.7%) across 6 sites. MIBI SPECT/CT scans were acquired and reported without the need for significant additional training. All scans were of adequate quality for interpretation. Sensitivity and specificity of MIBI SPECT/CT to detect cancer were 97.0% (95% CI 84.2–99.9%) and 53.8% (25.1–80.8%), respectively.

**Conclusion:**

MULTI-MIBI has demonstrated feasibility of recruitment to a diagnostic evaluation study for T1 renal masses. Preliminary estimates of diagnostic accuracy suggest that MIBI SPECT/CT could reduce the number of patients with benign tumours undergoing surgery without missing a significant number of patients with malignant disease, however these results are limited by the small sample size in this feasibility study and a larger definitive study is needed prior to adoption in practice.

**Supplementary Information:**

The online version contains supplementary material available at 10.1007/s00259-025-07525-3.

## Introduction

The standard management for patients diagnosed with a renal tumour is surgical excision [1]. Up to 30% of early-stage renal tumours are found to be benign on surgical histopathology, with overtreatment driven by a lack of accurate non-invasive diagnostic tools [2]. While localised renal cell carcinoma (RCC) usually requires treatment, benign tumours, most commonly renal oncocytoma, can be safely managed expectantly [3].

Pre-operative diagnostic tumour biopsy has been shown to reduce the proportion of patients with benign tumours undergoing surgery [4]. However inherent limitations of biopsy such as sampling only a small area of a potentially heterogenous tumour leads to the possibility of both over- and under-diagnosis and remains a barrier to uptake [5]. Investigation of new approaches to improve characterisation of incidentally detected small renal masses has been identified as a priority research gap [6].

In recent years, [^99m^Tc]Tc-sestamibi SPECT/CT (MIBI SPECT/CT) has been reported in several single-centre series to have high diagnostic accuracy to differentiate between RCC and oncocytic renal tumours, the most common type of benign renal tumour [7]. [^99m^Tc]Tc-sestamibi is a lipophilic cationic radiopharmaceutical with an affinity for mitochondria-rich cells and is well established in cardiac and parathyroid imaging. Oncocytic renal tumours possess high numbers of mitochondria compared to relatively mitochondria-poor RCCs, resulting in overtly different appearances on renal MIBI SPECT/CT imaging [8].

The current evidence base for renal MIBI SPECT/CT is limited by small single-centre series exclusively from academic institutions and it is not known if the results are generalisable to other settings. Our aim was to test feasibility of recruitment, local image acquisition and reporting to a prospective, multi-centre diagnostic test evaluation of MIBI SPECT/CT for T1 renal masses (MULTI-MIBI).

## Materials and methods

The MULTI-MIBI study was designed, conducted and reported according to STARD guidelines [9]. Full methods are described in the published protocol [10] (Ethics: UKHRA REC 20/YH/0279, registration ISRCTN12572202).

### Study design

**Fig. 1:**
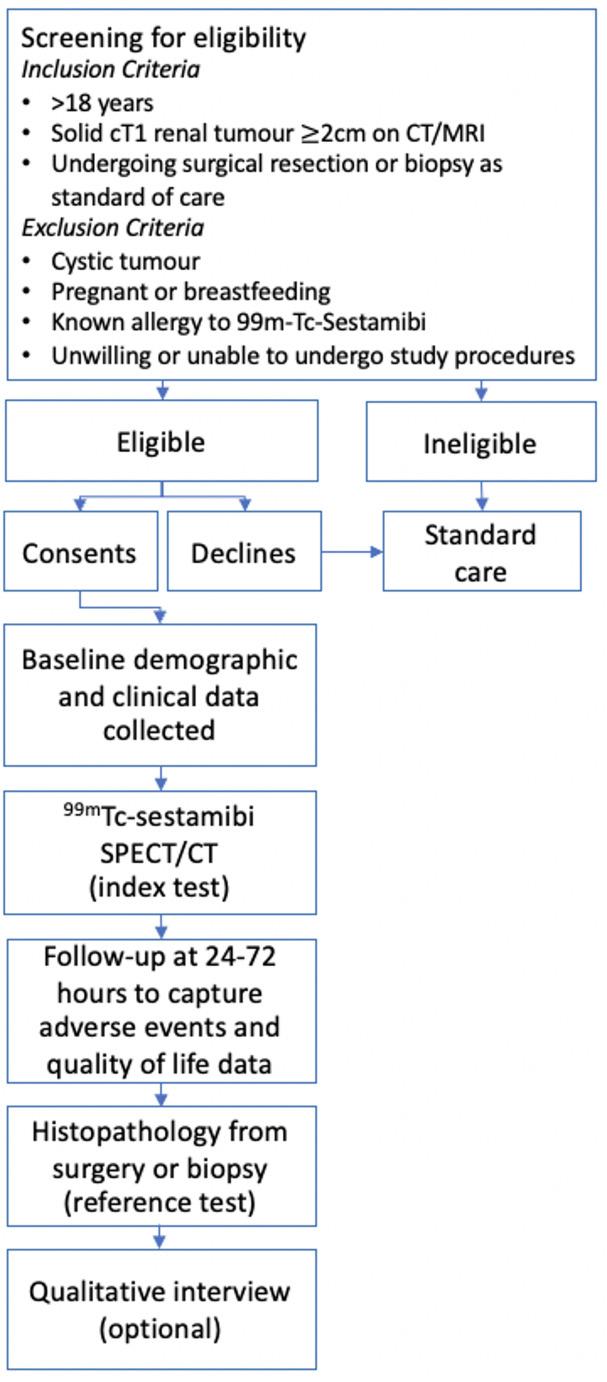
MULTI-MIBI study flow diagram

MULTI-MIBI was a prospective, feasibility, multi-centre diagnostic accuracy study of MIBI SPECT/CT for localised renal tumours. Study flow is illustrated in Fig. 1.

### Participants

Consecutive patients discussed at specialist urology/renal cancer multi-disciplinary team meetings between December 2022 and February 2024 were screened for eligibility. Eligibility criteria were adult patients ($\:\ge\:$18 years) of any gender with a clinical diagnosis of T1 solid renal tumour(s) of unknown pathological subtype, $\:\ge\:$2 cm in maximal tumour diameter in any dimension on standard care cross sectional imaging (CT or MRI) who were willing and able to provide informed consent. Patients were required to have surgery or renal tumour core biopsy planned as part of standard clinical care. Patients without histopathological confirmation of their tumour(s) histology i.e. those entering watchful waiting or active surveillance pathways without histology were excluded. Other exclusion criteria were cystic tumours, pregnant and breastfeeding patients, those with a known allergy to [^99m^Tc]Tc-sestamibi and those unwilling or unable to undergo the study procedures.

The intended sample size was 50 patients. Patients who accepted and declined participation and clinicians involved in study activities were invited to a semi-structured interview to explore perceptions, facilitators and barriers to recruitment and adoption of MIBI SPECT/CT.

### Test methods

900 MBq of [^99m^Tc]Tc-sestamibi was injected intravenously in a single bolus, 75 min before SPECT/CT acquisition. The study protocol was pragmatic, allowing for image acquisition in line with local experience of MIBI SPECT/CT for other conditions, but with the field of view set from the liver dome to the pelvis. Suggested minimum requirements were that participating centres should have SPECT/CT systems with the following specifications: at least 2-slice helical diagnostic CT scanner, available low-energy all-purpose or low-energy high-resolution collimator, gamma camera or digital detector elements appropriate for 140-kEv photopeak acquisition, and manufacturer-derived iterative reconstruction that includes scatter and attenuation correction. Supplementary Table 1 outlines the equipment and imaging protocols used during the study.

MIBI SPECT/CT scans were reported locally by experienced nuclear medicine clinicians/radiologists who received training via a half-day webinar that covered the principles of image interpretation, guided examples and ‘hands-on’ practice with a cloud-based training dataset, supported by international expert faculty.

Results of MIBI SPECT/CT were interpreted according to convention in cancer diagnostic test studies whereby a ‘positive’ result suggests cancer and a ‘negative’ result suggests benign disease. MIBI SPECT/CT scans were reported qualitatively according to pre-specified definitions as follows: in comparison to the normal renal parenchyma of the ipsilateral kidney, the tumour is (a) non-avid (no visible uptake in tumour, suggestive of cancer considered a positive result) (b) avid (visible uptake in tumour, suggestive of a benign tumour considered a negative result) (c) indeterminate. Quantitative assessments were also made, including relative maximum uptake ratio in the tumour compared to the ipsilateral normal renal parenchyma. Pseudonymised MIBI SPECT/CT images were transferred for central review at the lead site and discordant reports resolved by discussion and consensus. Local and central reporting was blinded to clinical information and the result of the histopathology reference test. Local reports were used in primary analyses.

Histopathology from surgery and/or biopsy formed the reference standard. In the case of a non-diagnostic biopsy the patient was offered a second attempt, according to local practice.

Histopathological reporting of both biopsy and surgical samples was performed by qualified pathologists at collaborating sites in accordance with the current World Health Organisation classification system for renal tumours [11], as per standard care. Pathologists were blinded to the MIBI SPECT/CT result. Pseudonymised diagnostic slides and/or blocks were transferred to the lead site for central review by a specialist uro-pathologist.

### Analysis

Recruitment was calculated as a proportion of those screened, eligible and approached along with 95% confidence intervals. Recruitment rate was also reported as median cases/month and interquartile range.

Diagnostic accuracy of MIBI SPECT/CT was estimated by generating 2 × 2 tables for both avid and non-avid qualitative assessment, and relative radiotracer uptake ratio > 0.6 for external validation of a pre-defined threshold from the literature [12]. Visual inspection of relative uptake ratios was performed for exploratory assessment at different thresholds. Diagnostic accuracy of MIBI SPECT/CT was calculated in terms of sensitivity, specificity, positive and negative predictive values along with their 95% confidence intervals. The prevalence of renal oncocytoma and other histology subtypes was reported as a proportion of all cases.

The proportion of participants with invalid MIBI SPECT/CT results e.g. due to technical failure was reported, as well as the proportion of valid but inconclusive results. Invalid and indeterminate results were excluded from further analyses [13]. Inter-rater agreement of qualitative assessment of [^99m^Tc]Tc-sestamibi SPECT/CT scans was reported using percentage agreement and Gwet’s first-order agreement coefficient [14].

### Qualitative interviews

Approached patients who agreed and declined to participate in MULTI-MIBI and clinicians involved in study activities were invited to participate in an optional semi-structured qualitative interview, exploring barriers and facilitators to recruitment, site set up and study activities. Study interviews were conducted using rapid feedback loop design [15], whereby data are analysed in parallel to collection using RREAL sheets [16]. Emerging findings were fed back to the study team in real time at monthly trial management group meetings.

## Results

**Fig. 2:**
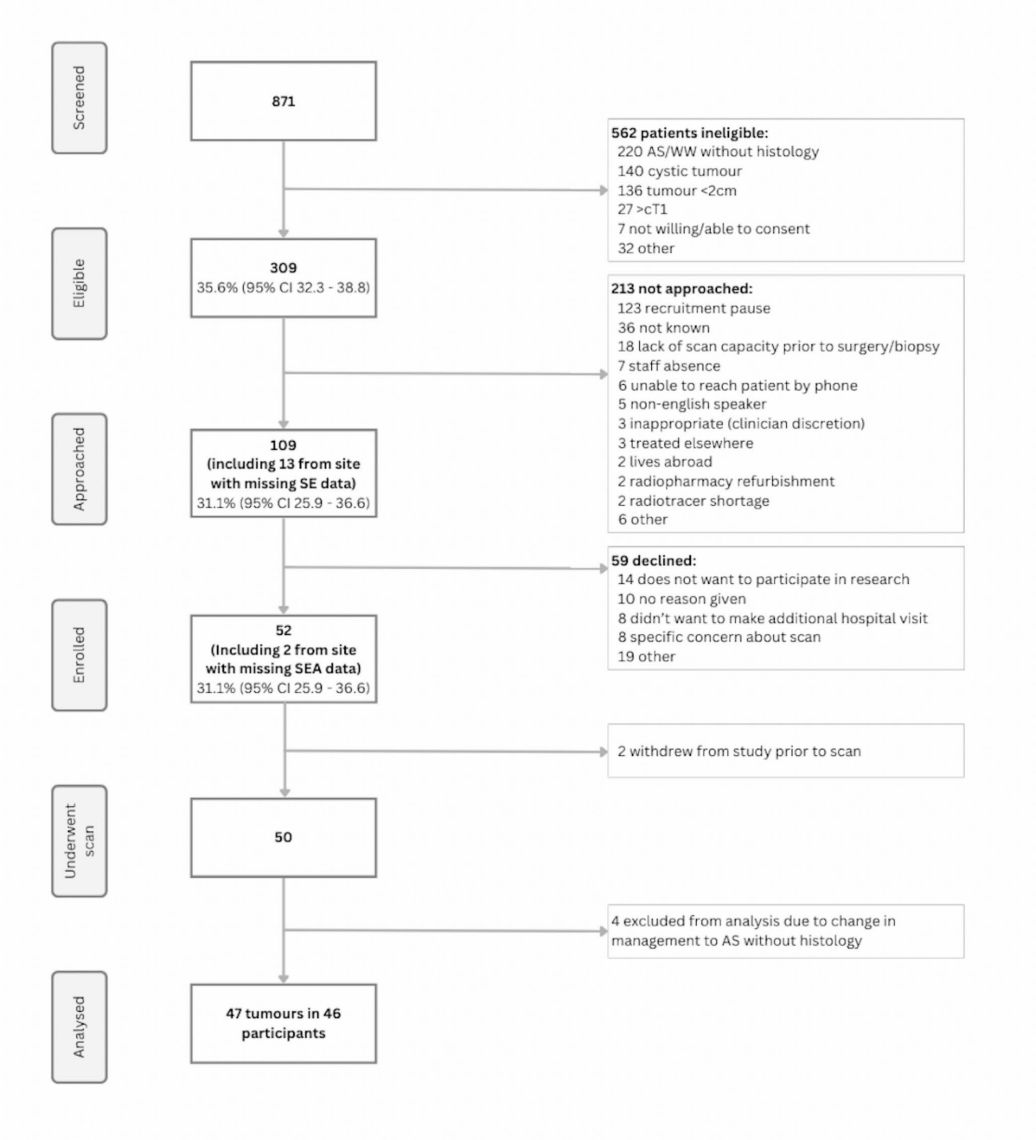
Screened, eligible, approached and enrolled (SEAR) participants during the 15-month study period across six UK sites, with reasons for exclusion. 95% CI = 95% confidence interval, AS = active surveillance, WW = watchful waiting

Over a planned recruitment period of 15 months (December 2022-February 2024), 50 patients underwent MIBI SPECT/CT. Screened, eligible, approached and enrolled data is reported in Fig. 2. All six sites opened and recruited (range 2–25 participants). The lead site paused recruitment for the final 6 months of the recruitment period to test resilience, and recruitment completed 3 weeks ahead of schedule. The mean recruitment was 3.3 patients/month (SD 2.1).

The study population was representative of the general UK kidney cancer population in terms of gender (70% male), age (mean 66 years [IQR 56–74]) and ethnicity (76% white, 8% black, 8% Asian, 4% mixed, 4% other). The study population was socioeconomically diverse, with participants recruited from every decile of the index of multiple deprivation (IMD) [17].

**Fig. 3:**
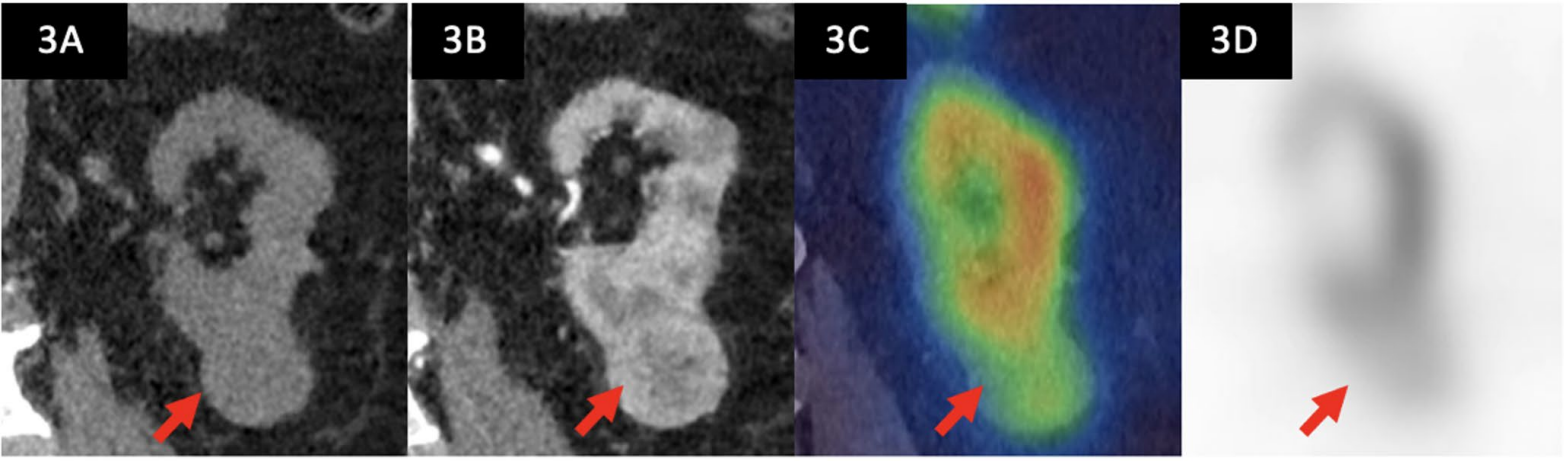
Diagnostic imaging of a 36mm left lower pole tumour indicated by the red arrow, diagnosed on biopsy as a renal oncocytoma. This tumour was interpreted as avid relative to the normal parenchyma of the ipsilateral kidney **A**) coronal non-contrast CT of the left kidney **B**) corresponding contrast-enhanced CT demonstrating a solid, enhancing, heterogenous tumour **C**) coronal [^99m^Tc]Tc-sestamibi SPECT-CT images shows radiotracer uptake, similar to that of the surrounding renal parenchyma **D**) corresponding coronal SPECT image shows [^99m^Tc]Tc-sestamibi uptake [Computed tomography = CT, Single photon emission computed tomography = SPECT]

**Fig. 4:**
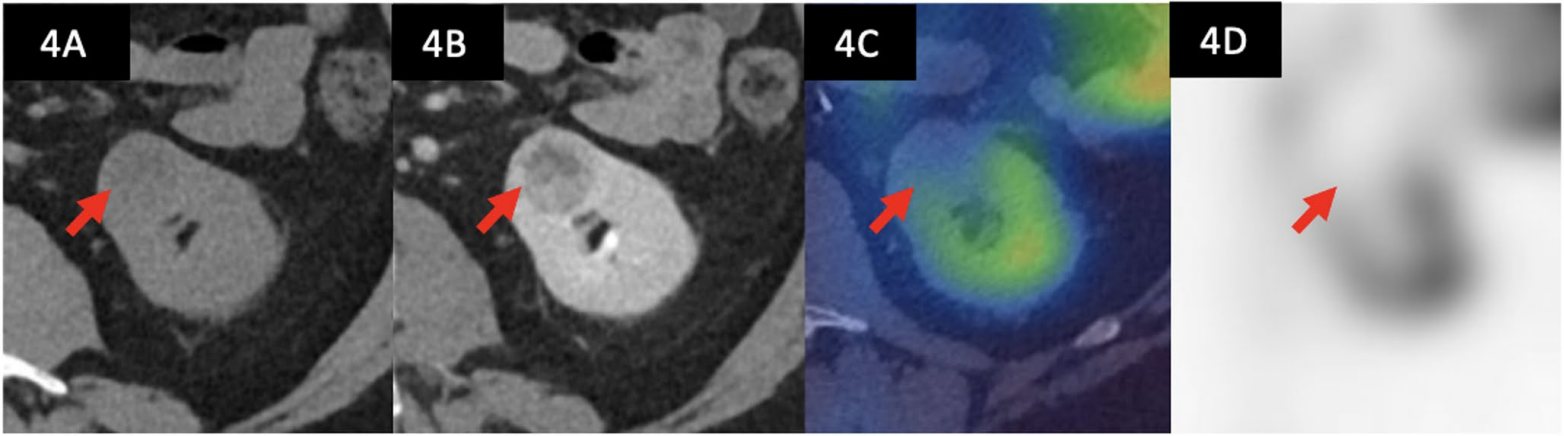
Diagnostic imaging of a 24 mm left anterior tumour indicated by the red arrow, diagnosed on biopsy as a clear cell renal cell carcinoma. This tumour was interpreted as photopaenic relative to the normal parenchyma of the ipsilateral kidney **A**) axial non-contrast CT of the left kidney **B**) corresponding contrast-enhanced CT demonstrating a solid, enhancing, heterogenous tumour (C) axial [^99m^Tc]Tc-sestamibi SPECT-CT images shows no radiotracer uptake in the region of the tumour (D) corresponding axial SPECT image shows no [^99m^Tc]Tc-sestamibi uptake [Computed tomography = CT, Single photon emission computed tomography = SPECT]

Four participants did not have evaluable histology due to changes to planned clinical care (active surveillance without histological diagnosis) therefore 46 out of 50 patients scanned, with 47 tumours, were included in the analysis. Median tumour diameter was 30 mm (IQR 25–42) and clinical stage cT1a (72%) cT1b (28%). Cancer prevalence was 70.2% (40.4% clear cell, 21.3% papillary, 2.1% chromophobe, 2.1% clear cell papillary, 4.2% RCC not otherwise specified) and benign disease 29.8% (19.1% oncocytoma, 6.4% angiomyolipoma, 4.2% other oncocytic tumour). All MIBI SPECT/CT scans were considered valid (of sufficient quality for interpretation), and no repeat MIBI SPECT/CT scans were required. There was one grade 1 adverse event (temporary exacerbation of rotator cuff pain from holding arms above head for study scan). On MIBI SPECT/CT, 38 tumours were non-avid, 8 avid and 1 indeterminate. Example study images are shown in Figs. 3 and 4. Median time from index test (MIBI SPECT/CT) to reference test (histopathology) was 12.5 days (IQR 6–24 days).

**Fig. 5:**
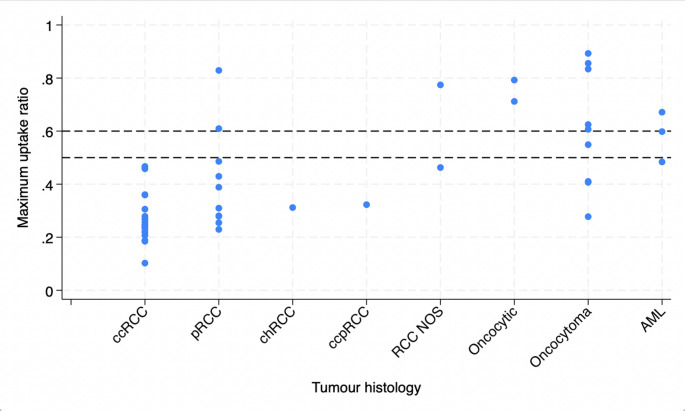
Maximum uptake ratio in a spherical region of interest drawn within the tumour relative to the normal ipsilateral renal parenchyma, by histological subtype. Malignant: clear cell renal cell carcinoma (ccRCC), papillary renal cell carcinoma (pRCC), chromophobe renal cell carcinoma (chRCC), clear cell papillary renal cell carcinoma (ccpRCC), renal cell carcinoma not otherwise specified (RCC NOS); benign: oncocytic tumours of low malignant potential, oncocytoma, angiomyolipoma (AML)

Local qualitative assessment of MIBI SPECT/CT and tumour histology is reported in Table 1, resulting in estimates of diagnostic accuracy of MIBI SPECT/CT to detect cancer as follows: sensitivity 97.0% (95% CI 84.2–99.9%), specificity 53.8% (25.1–80.8%), positive predictive value 84.2% (68.7–94%), negative predictive value 87.5% (47.3–99.7%). Inter-rater agreement was almost perfect: Gwet’s first-order coefficient = 0.9408 (95% CI 0.64-1). Quantitative assessment of relative uptake ratio in a region of interest within the tumour relative to the surrounding renal parenchyma by tumour subtype is shown in Fig. 5. Estimates of diagnostic accuracy of MIBI SPECT/CT based on quantitative analyses is reported in Table 2.

**Table 1:** Tumour pathology and MIBI SPECT/CT results according to qualitative assessment by the local reporting clinician

Histology (*N* = 47)	MIBI SPECT/CT non-avid, suggests cancer	MIBI SPECT/CT avid, suggests benign	Indeterminate
Cancer			
Clear cell RCC	19	0	0
Papillary RCC	9	1	0
Chromophobe RCC	1	0	0
Clear cell papillary RCC	1	0	0
RCC NOS	2	0	0
Benign			
Oncocytoma	4	4	1
Angiomyolipoma	2	1	0
Other oncocytic tumour	0	2	0

**Table 2:** Estimates of diagnostic accuracy along with 95% confidence intervals for MIBI SPECT/CT to detect malignancy based on quantitative analysis of the tumour maximum uptake relative to the normal ipsilateral renal parenchyma

A) Malignant v benign % (95% CI)	< 0.6 uptake ratio (pre-specified)	< 0.5 uptake ratio (exploratory)
Sensitivity	90.9 (75.7–98.1)	90.9 (75.7–98.1)
Specificity	57.1 (28.9–82.3)	71.4 (41.9& − 91.6%)
PPV	83.3 (67.2–93.6)	88.2 (72.5–96.7)
NPV	72.7 (39–94)	76.9 (46.2–95%)

Ten clinicians (3 urologists, 2 research nurses, 5 nuclear medicine technologists/clinicians) and nineteen patients participated in qualitative interviews (16 who had a MIBI SPECT/CT scan and three who had declined or withdrawn). Key facilitators to patient recruitment were the pragmatic, patient-focused study activities that required just one additional hospital visit, patient altruism and a desire for development of a non-invasive test. Barriers included challenges with scheduling the scan before expedited surgery or biopsy for suspected malignancy, concerns about radiation exposure raised in participant information leaflets and the study offering no benefit to individual participants. Nuclear Medicine technologists commented that the ease of delivering study scans was a facilitator with sites scheduling MULTI-MIBI participants’ scans alongside MIBI SPECT/CT scans performed for other routine clinical indications. Clinicians who reported MIBI SPECT/CT scans as part of the trial expressed positive experience and satisfaction with the training workshop for image interpretation.

## Discussion

The MULTI-MIBI study has demonstrated feasibility of multi-site recruitment of participants to a prospective diagnostic accuracy study of MIBI SPECT/CT for the evaluation of solid cT1 renal tumours. The study met its recruitment target of 50 patients 3 weeks ahead of schedule, with the lead site paused for 6 months of the 15-month recruitment period. This demonstrates resilience to closure and/or recruitment pauses at individual sites, including the lead site.

Single centre studies of similar design have successfully recruited [18–22], however this is the first multi-centre study, and the first in the UK besides pump-priming work [23]. MULTI-MIBI has demonstrated feasibility for nuclear medicine departments across the UK to acquire and interpret MIBI SPECT/CT studies without the need for significant additional training. Near-perfect inter-rater agreement between local and central reviewers suggests that diagnostic accuracy of the [^99m^Tc]Tc-sestamibi SPECT/CT does not vary by reporter.

Early reports of MIBI SPECT/CT for renal masses considered an avid SPECT/CT suggestive of oncocytoma to be ‘positive’. Our approach was to keep convention with terminology used for the majority of cancer diagnostic tests, whereby a test suggestive of cancer is ‘positive’ and a test suggestive of benign disease is ‘negative’. This allows for more intuitive interpretation of measures of diagnostic accuracy and comparison with tests used in other settings.

A recent meta-analysis reported sensitivity and specificity of MIBI SPECT/CT for T1 renal tumours of 88.6% (95% CI 82.7%– 92.6%) and 77.0% (95% CI 63.0–86.9%), respectively using the same definitions of a positive or negative test as in MULTI-MIBI [7]. While MULTI-MIBI was not powered to make precise estimates of diagnostic accuracy, the estimated sensitivity in MULTI-MIBI was higher, and the specificity lower than is reported in the previous systematic review. The demographics of the study population did not appear sufficiently different from previous reports to account for the observed difference in measures of diagnostic accuracy and similar acquisition protocols were utilised in MULTI-MIBI [24, 25]. While the discrepancy could be due to the small sample size in this feasibility study, we also considered the diagnostic threshold used to define MIBI SPECT/CT as positive or negative in MULTI-MIBI compared to previously published studies. Our qualitative definition of radiotracer uptake relative to the normal ipsilateral kidney parenchyma was in keeping with early reports [25], however differed to a later description by Campbell et al. that suggests *any* radiotracer uptake in the region of the tumour can suggest oncocytoma [24]. Adopting a different diagnostic threshold may have resulted in correct identification of more oncocytomas (higher specificity) though likely at the expense of sensitivity, exemplified in the quantitative threshold analysis (Fig. 5). Future studies should consider the role of MIBI SPECT/CT in the diagnostic pathway when considering if sensitivity or specificity is prioritised when selecting a diagnostic threshold.

The single RCC misclassified as negative on MIBI SPECT/CT due to radiotracer uptake in the region of the tumour was a low grade papillary RCC with eosinophilic cytoplasm. The term ‘eosinophilic’ reflects the pink appearance on light microscopy of mitochondria-rich cells when stained with hematoxylin and eosin and may accounts for the high radiotracer uptake in this tumour [26]. While most eosinophilic renal tumours have an indolent clinical course, and for clinical purposes can be grouped together as ‘low-risk oncocytic tumours’ [27], there exist some rare but poor-prognosis or hereditary tumours e.g. fumarate hydratase deficient RCC or SDHB deficient RCC that are important to diagnose. As far as we are aware there are no reports of these rare tumours imaged with MIBI SPECT/CT in the literature, however, it is plausible that they may show avidity.

Approximately a third of participants in MULTI-MIBI had benign tumours, reflective of population level data [2], resulting in less precise estimates of specificity than sensitivity. This will inform sample size calculations in follow on work.

According to Grading of Recommendations Assessment, Development and Evaluation (GRADE) the ideal body of evidence for evaluating diagnostic studies would include studies that make a direct comparison of test strategies and the downstream resulting interventions and consequences as patient-important outcomes [28, 29]. A large-scale definitive study is needed to determine precise estimates of diagnostic accuracy for MIBI SPECT/CT and would ideally make a comparison with other diagnostic approaches, report the downstream consequences of managing patients according to MIBI SPECT/CT results, and evaluate cost effectiveness.

## Conclusions

In summary, MULTI-MIBI has shown that recruitment to a large, multi-centre diagnostic test evaluation of MIBI SPECT/CT for the non-invasive characterisation of cT1 renal tumours is feasible. Local reporting is possible without significant additional training. A full trial assessing the role and cost-effectiveness of MIBI for T1 renal tumours is now planned.

## Supplementary Information

Below is the link to the electronic supplementary material.

ESM1 (16.0 KB)

## Data Availability

The datasets generated during and/or analysed during the current study are available from the corresponding author on reasonable request.
