# Supplementary material for: [99mTc]Tc-sestamibi SPECT/CT for the diagnosis of kidney tumours: a multi-centre feasibility study (MULTI-MIBI Study)
Source: Eur J Nucl Med Mol Imaging. 2025 Sep 23;53(3):1719–28. doi: 10.1007/s00259-025-07525-3 (PMC12860857; doi:10.1007/s00259-025-07525-3)
Supplement: Supplementary file 1 — ESM1 (16.0 KB) [file 259_2025_7525_MOESM1_ESM.docx]

|  | Site 1 | Site 2 | Site 3 | Site 4 | Site 5 | Site 6 |
| --- | --- | --- | --- | --- | --- | --- |
|  | Siemens Symbia Intevo Bold | GE healthcare  CZT 870 | GE Healthcare Discovery 670 / 670 Pro | GE Optima NM/CT640 | Siemens Symbia Intevo Bold | GE Discovery 670 |
| SPECT parameters | | | | | | |
| Zoom | 1 | 1 | 1 | 1 | 1 | 1 |
| Number of Views | 60 (30/detector) | 60 (30/detector) | 120 (60/detector) | 60 (30/detector) | 128 (64/detector) | 120 (60/detector) |
| Energy | 140 keV +/- 15% | 140.5 keV +/- 15% | 140.5 keV +/- 10% | 140 keV | 140 keV +/- 15% | 140.5 keV ± 10% |
| Camera preset | Tc-99m-NMG | Tc99m | Tc99m | Tc99m | Tc99m | Tc99m |
| Matrix size | 128x128 | 128x128 | 128x128 | 128x128 | 128x128 | 256 x 256 |
| Detectors | Both | Both | Both | Both | Both | Both |
| Collimation | LEHR | WEHR 45 | LEHR | LEHR | LEHR | LEHR |
| Degrees of rotation | 360 (180 per detector) step and shoot | 360 (180 per detector) step and shoot | 360 (180 per detector) step and shoot | 360 (180 per detector) step and shoot | 360 (180 per detector) step and shoot | 360 (180 per detector) Step & Shoot |
| Time per view (s) | 28 | 20 | 15 | 30 | 20 | 20 |
| CT parameters | | | | | | |
| Orientation | Head out | Feet first supine | Head first supine | Head first supine | Head out | Feet first supine |
| kV | 130 | 120 | 120 | 120 | 140 | 120 |
| CT recon | Standard | Standard | Standard | Standard | Standard | FBP |
| Matrix |  | 512x512 | 512x512 | 512x512 | 512x512 | 256 |
| Pitch | 1.2 |  |  | 1.25 |  | 1.375 |
| Slice thickness (mm) | 2 | 2.5 | 1.25 | 2.5 | 1.5 | 2.5 |
| mAs | 65 | Smart mA (min 30 max 200) | Smart mA (min 30 max 200) | 20 |  | Smart mA (max mA 120) |

LEHR = Low energy high resolution, WEHR = wide energy high resolution
